# Supplementary material for: EnzML: multi-label prediction of enzyme classes using InterPro signatures
Source: BMC Bioinformatics. 2012 Apr 25;13:61. doi: 10.1186/1471-2105-13-61 (PMC3483700; doi:10.1186/1471-2105-13-61)
Supplement: Addtional file 5 — The Java code to format the data files, evaluate and predict. The file enzml_java_code.tar.gz contains the Java code used to format database data to ARFF and XML formats, to execute cross and train-test (jackknife) evaluations and to record evaluation results to database. More information is included in the readme.txt file and the Javadoc files. The code can be used with a MySQL database. To use a different database software, other JDBC drivers might be required. [file 1471-2105-13-61-S5.gz › java_code/utils/doc/edu/cornell/lassp/houle/RngPack/RandomSeedable.html]

RandomSeedable


---


|  |  |  |
| --- | --- | --- |
| **Overview** \| **Package** \| **Class** \| **Use** \| **Tree** \| **Deprecated** \| **Index** \| **Help** | |  |
| **PREV CLASS**   **NEXT CLASS** | **FRAMES**    **NO FRAMES**     **All Classes** |  |
| SUMMARY: NESTED \| FIELD \| CONSTR \| METHOD | DETAIL: FIELD \| CONSTR \| METHOD |  |


---


## edu.cornell.lassp.houle.RngPack Class RandomSeedable

```
java.lang.Object
  cern.colt.PersistentObject
      edu.cornell.lassp.houle.RngPack.RandomElement
          edu.cornell.lassp.houle.RngPack.RandomSeedable
```

**All Implemented Interfaces:**: java.io.Serializable, java.lang.Cloneable

**Direct Known Subclasses:**: RandomEngine, Ranecu, Ranlux, Ranmar

---

``` public abstract class RandomSeedable extends RandomElement ```

`RandomSeedable` is an abstract class that extends the
`RandomElement` class to include the ability to automatically
generate a valid `long` seed from the clock. Thus it provides a
consistent interface for seeding interchangable generators. It is reccomended
that a `RandomSeedable` have a constructor that takes a
`long` for a seed. For example, if you write a generator called
`ReallyRandom`, you want to be able to do

```
 long seed = ReallyRandom.ClockSeed();
 RandomSeedable e = new ReallyRandom(seed);
```

this makes it convenient to keep a copy of the seed in case you want to
restart the generator with the same seed some time in the future.

If one is going to use a long to generate a smaller seed by taking
`Clockseed()` modulus another number, we reccomend that you use a
prime number; this ensures that the generator would have the maximum "period"
if it were started at regular issues, for instance, by a batch job. See
`Ranmar` for an example.

Source
code  is available.

**Version:**
:   1.0

**Author:**
:   Paul Houle  (E-mail:
    houle@msc.cornell.edu)

**See Also:**: `Ranecu`, `Ranlux`, `Ranmar`, Serialized Form

---

**Constructor Summary**

`RandomSeedable()`


| **Method Summary** | |
| --- | --- |
| `static long` | `ClockSeed()`             Return a long integer seed calculated from the date. |
| `static long` | `ClockSeed(java.util.Date d)`             Return a long integer seed given a date |

**Methods inherited from class edu.cornell.lassp.houle.RngPack.RandomElement**

`choose, choose, gaussian, gaussian, powlaw, raw, raw, raw, uniform`

**Methods inherited from class cern.colt.PersistentObject**

`clone`

**Methods inherited from class java.lang.Object**

`equals, getClass, hashCode, notify, notifyAll, toString, wait, wait, wait`

**Constructor Detail**

### RandomSeedable

```
public RandomSeedable()
```


**Method Detail**

### ClockSeed

```
public static long ClockSeed()
```

:   Return a long integer seed calculated from the date. Equivalent to
    `ClockSeed(new Date());

    :   Returns:: a long integer seed`

---


### ClockSeed

```
public static long ClockSeed(java.util.Date d)
```

:   Return a long integer seed given a date

    :   **Parameters:**: `d` - a date **Returns:**: a long integer seed


---


|  |  |  |
| --- | --- | --- |
| **Overview** \| **Package** \| **Class** \| **Use** \| **Tree** \| **Deprecated** \| **Index** \| **Help** | |  |
| **PREV CLASS**   **NEXT CLASS** | **FRAMES**    **NO FRAMES**     **All Classes** |  |
| SUMMARY: NESTED \| FIELD \| CONSTR \| METHOD | DETAIL: FIELD \| CONSTR \| METHOD |  |


---
